# Evolution of longevity improves immunity in *Drosophila*

**DOI:** 10.1002/evl3.89

**Published:** 2018-11-12

**Authors:** Daniel K. Fabian, Kathrin Garschall, Peter Klepsatel, Gonçalo Santos‐Matos, Élio Sucena, Martin Kapun, Bruno Lemaitre, Christian Schlötterer, Robert Arking, Thomas Flatt

**Affiliations:** ^1^ Centre for Pathogen Evolution, Department of Zoology University of Cambridge Cambridge United Kingdom; ^2^ Institut für Populationsgenetik Vetmeduni Vienna Vienna Austria; ^3^ Vienna Graduate School of Population Genetics Vienna Austria; ^4^ Department of Ecology and Evolution University of Lausanne Lausanne Switzerland; ^5^ Institute of Zoology Slovak Academy of Sciences 845 06 Bratislava Slovakia; ^6^ Instituto Gulbenkian de Ciência Oeiras Portugal; ^7^ Departamento de Biologia Animal Faculdade de Ciências da Universidade de Lisboa Lisboa Portugal; ^8^ Global Health Institute School of Life Sciences, EPFL Lausanne Switzerland; ^9^ Department of Biological Sciences Wayne State University Detroit Michigan; ^10^ Department of Biology University of Fribourg Fribourg Switzerland

**Keywords:** Aging, *Drosophila*, evolve, immunity, longevity, resequence

## Abstract

Much has been learned about the genetics of aging from studies in model organisms, but still little is known about naturally occurring alleles that contribute to variation in longevity. For example, analysis of mutants and transgenes has identified insulin signaling as a major regulator of longevity, yet whether standing variation in this pathway underlies microevolutionary changes in lifespan and correlated fitness traits remains largely unclear. Here, we have analyzed the genomes of a set of *Drosophila melanogaster* lines that have been maintained under direct selection for postponed reproduction and indirect selection for longevity, relative to unselected control lines, for over 35 years. We identified many candidate loci shaped by selection for longevity and late‐life fertility, but – contrary to expectation – we did not find overrepresentation of canonical longevity genes. Instead, we found an enrichment of immunity genes, particularly in the Toll pathway, suggesting that evolutionary changes in immune function might underpin – in part – the evolution of late‐life fertility and longevity. To test whether this genomic signature is causative, we performed functional experiments. In contrast to control flies, long‐lived flies tended to downregulate the expression of antimicrobial peptides upon infection with age yet survived fungal, bacterial, and viral infections significantly better, consistent with alleviated immunosenescence. To examine whether genes of the Toll pathway directly affect longevity, we employed conditional knockdown using in vivo RNAi. In adults, RNAi against the *Toll* receptor extended lifespan, whereas silencing the pathway antagonist *cactus*‐–causing immune hyperactivation – dramatically shortened lifespan. Together, our results suggest that genetic changes in the age‐dependent regulation of immune homeostasis might contribute to the evolution of longer life.

**Impact Summary**

Despite much progress in our understanding of the genetic basis of aging, mainly from studying large‐effect mutants, little is known about natural variants that contribute to the evolution of lifespan and related fitness traits. To identify the mechanisms by which longevity evolves, we sequenced a set of *D. melanogaster* populations that have been undergoing selection for late‐life reproduction and postponed senescence, relative to unselected controls, for over 35 years. Instead of an enrichment of evolutionary changes in previously identified “canonical” longevity genes, we found an enrichment of genetically diverged immunity genes, suggesting that variation in immune function contributes to the evolution of lifespan and late‐life fertility. To test this hypothesis, we employed immunity assays: long‐lived flies survived infections better and showed altered age‐dependent immune gene expression as compared to control flies. Using in vivo RNAi we confirmed that reduced expression of immune genes extends lifespan while immune overactivation is strongly detrimental.

Despite major progress in our understanding of the genetic basis of aging and life history, especially in model organisms such as yeast, *C. elegans, Drosophila*, and mice (Guarente and Kenyon 2000; Partridge and Gems 2002; Tatar et al. 2003; Guarente et al. 2008; Kenyon 2010; Flatt and Heyland 2011), the identity and effects of naturally segregating polymorphisms that affect variation in lifespan and correlated fitness traits and which might thus underpin the evolution of longevity and life history remain poorly understood to date (De Luca et al. 2003; Pasyukova et al. 2004; Carbone et al. 2006; Flatt and Schmidt 2009; Paaby et al. 2014; Carnes et al. 2015; Flatt and Partridge 2018).

Several major evolutionarily conserved pathways that regulate lifespan and correlated fitness traits, including insulin/insulin‐like growth factor 1 signaling (IIS), have been identified using analyses of large‐effect mutants and transgenes in the laboratory (Partridge and Gems 2002; Tatar et al. 2003; Kenyon 2010), but to what extent genes in these “canonical” pathways harbor segregating alleles that affect lifespan is mostly unknown (Flatt and Schmidt 2009; Paaby et al. 2014; Carnes et al. 2015; Flatt and Partridge 2018). For instance, only few studies to date have identified functional effects of segregating IIS polymorphisms upon lifespan and correlated life‐history traits in populations of *Drosophila* (Paaby et al. 2010, 2014; Remolina et al. 2012) or which contribute to longevity in human centenarians (Suh et al. 2008; Willcox et al. 2008; Flachsbart et al. 2017; Joshi et al. 2017).

Here, we take advantage of a >35‐year‐long laboratory selection experiment for late‐life fertility and increased lifespan in *Drosophila melanogaster*, first published by Luckinbill and colleagues in 1984 (Luckinbill et al. 1984; also see Luckinbill and Clare 1985; Arking 1987), to analyze the genomic footprints underlying the evolution of delayed reproduction and postponed aging. In this long‐term selection experiment, replicate lines derived from an outbred base population have been selected for late‐life fertility and–indirectly–for increased lifespan by breeding only from flies that survived and were fertile at a relatively old age. In contrast, unselected replicate control lines have been propagated across generations by breeding from flies with a random age at reproduction (for details see Supplementary methods). Selected flies in this experiment have evolved late‐life fertility and live ≈40–50% longer than unselected control flies, yet exhibit reduced early fecundity relative to the controls (see Supplementary methods). Thus, these selection lines are subject to a genetic trade‐off between late‐life performance (long life, late‐life fertility) and early fecundity, as is commonly observed in laboratory evolution experiments that directly or indirectly select for changes in *Drosophila* lifespan (Luckinbill et al. 1984; Rose 1984; Zwaan et al. 1995; Partridge et al. 1999; Stearns et al. 2000; Remolina et al. 2012).

The central finding from our genomic analysis of this selection experiment is that evolutionary changes in innate immunity contribute to the evolution of late‐life performance in fruit flies, probably by improving age‐dependent immune homeostasis. Although still little is understood about the mechanistic interplay between immunity and aging (Garschall and Flatt 2018), our analyses suggest that immune function is a major longevity assurance mechanism that can be targeted by selection on standing genetic variation.

## Results and Discussion

### POOL‐SEQ IDENTIFIES A GENOME‐WIDE SIGNATURE OF LONGEVITY

**Figure 1:**
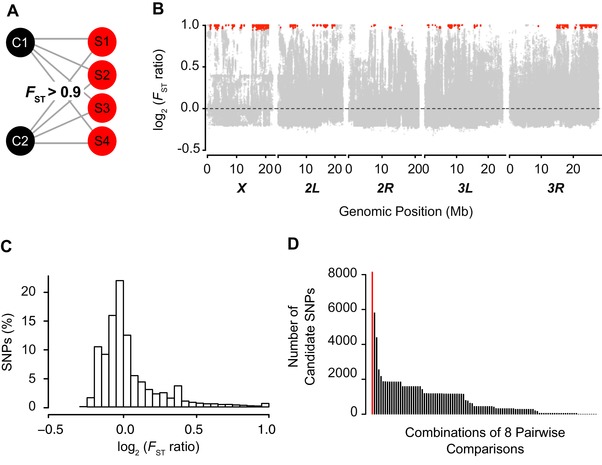
**Genomic response to longevity selection. (A)** Identification of longevity candidates. To identify candidate SNPs that have likely been shaped by selection for longevity we performed all eight pairwise *F*_ST_ comparisons between the two unselected control lines (C1, C2) and the four long‐lived selection lines (S1, S2, S3, S4). SNPs were defined to represent candidates if *F*_ST(selection vs. control)_ > 0.9 in all eight pairwise comparisons and if they showed significant allele frequency differentiation between the selection and control regime (Fisher's Exact test, Bonferroni *P* < 10^−9^). See Supplementary methods for details. Using this stringent *F*_ST_ outlier approach we identified 8205 candidate SNPs belonging to 868 genes. **(B)** Genomic “selection signal” relative to “noise.” To quantify the strength of genetic differentiation among the selection and control lines (“selection signal”) relative to differentiation within control or selection lines (“noise”) we calculated a “selection signal”‐to‐noise ratio. This ratio provides a measure of average *F*_ST_ differentiation among the selection versus control regime relative to *F*_ST_ differentiation within regimes (see Supplementary methods). Positive values of this log_2_ *F*_ST_ ratio indicate larger differentiation among regimes relative to within regimes, thus representing a “signal” of selection. The genome‐wide distribution of this ratio has a mode ≈ 0, indicating equal differentiation among and within regimes. Only a very small fraction of SNPs has a ratio ≈ 1 that would indicate complete allelic fixation (*F*_ST_ = 1) among regimes, without any differentiation within regimes. We focused our genomic analysis on candidate SNPs that represent extreme *F*_ST_ outliers with a ratio of ≈ 0.9. **(C)** Genomic locations of candidate SNPs. log_2_ *F*_ST_ ratio as function of genomic position on chromosomal arms *X*, *2L*, *2R*, *3L*, and *3R*. Candidate SNPs are shown in red and noncandidates (i.e., nonsignificant genomic background) in gray. Note the vast excess of highly differentiated SNPs in the selection versus control regime comparisons (values > 0), in marked contrast to the much weaker differentiation within the control and selection regimes (values < 0). **(D)** Number of candidate SNPs in different combinations of eight pairwise comparisons. To define candidate SNPs we performed all possible eight pairwise comparisons between two control and four selection lines and used a stringent *F*_ST_ outlier approach (see Supplementary methods). This yielded 8205 candidate SNPs (red bar) belonging to 868 candidate genes. To verify that this number of candidate SNPs is not due to chance we applied our candidate criteria to all 6435 possible sets of eight pairwise comparisons; out of these combinations only one set is biologically informative in terms of inferring selection, that is the set of all eight pairwise control versus selection comparisons (see Supplementary methods). No combination of eight pairwise comparisons yielded as many candidate SNPs as this “true” set of comparisons (red bar), with a probability that the “true” number of candidate SNPs is due to chance of *P* ≈ 1.6 × 10^−4^.

To characterize the genomic signature of longevity we used next‐generation pool‐sequencing (Pool‐seq) (Schlötterer et al. 2014) to obtain genome‐wide allele frequency estimates from four long‐lived selection lines and two unselected control lines after ≥ 144 generations of selection (see Supplementary methods for details). We identified candidate SNPs by comparing allele frequency differentiation between the selection and control regimes with a stringent *F*_ST_ outlier approach (Lewontin and Krakauer 1973; Akey 2009) (Fig. 1A,B). The majority of SNPs (62.2%) showed no or less differentiation between the selection versus control regime as compared to differentiation within these regimes (selection signal‐to‐noise ratio ≤ 0; Fig. 1B,C). We defined SNPs as candidates if they showed very strong, consistent and significant differentiation in all eight pairwise comparisons between the four selection and two control lines (signal‐to‐noise ratio ≈ 0.9; *F*_ST(selection vs. control)_ > 0.9; Bonferroni‐corrected Fisher's exact test: *P* < 10^−9^) (Fig. 1A,B,C). Using this approach, we identified 8205 candidate SNPs in 868 genes distributed across the entire genome (Fig. 1B; Table S1; genes were defined as the sequence between the ends of the 5’ and 3’ UTRs plus 1 kb up‐ and downstream; also see Supplementary methods). Candidate loci appeared to cluster non‐randomly in specific genomic regions, suggesting pervasive polygenic selection and/or indirect selection due to “hitchhiking” (“genetic draft”) (Fig. 1B; Table S1). To further validate our set of longevity candidate SNPs and to exclude false positives due to randomness, for example because of genetic drift, we used a combinatorial approach (see Supplementary methods). We found that–when applying our stringent candidate criteria – it is highly unlikely (*P* ≈ 1.6 × 10^−4^) that this large number of candidate SNPs arose by chance (Fig. 1D).

### LONGEVITY CANDIDATE GENES EXHIBIT GENETIC PARALLELISM

While some mechanisms of longevity are evolutionarily conserved (“shared”) among species and thus “public,” for example insulin/insulin‐like growth factor 1 signaling (IIS), most others are likely to be lineage‐specific and thus ‘private’ (Martin et al. 1996; Partridge and Gems 2002; McElwee et al. 2007). Similarly, at the intraspecific level, parallel and convergent evolution in independent populations might result in the repeated use of the same genes underlying a given trait (“gene reuse”) (Conte et al. 2012), but to what extent this might be the case for longevity remains unclear. Addressing this question might give insights into the predictability of the evolution of lifespan at the genetic level (Stern and Orgogozo 2008; Conte et al. 2012).

To examine how frequently the same genes are used by different populations during the evolution of late‐life fertility and longevity, we compared our list of candidate genes to those from two other “Evolve and Resequence” studies of *Drosophila* longevity and correlated life‐history traits (Remolina et al. 2012; Carnes et al. 2015). The study by Carnes et al. (2015) provides a genomic analysis of an independent long‐term selection experiment by Rose (Rose 1984) similar in duration to ours (Luckinbill et al. 1984), with both selection experiments first published back‐to‐back in 1984. The other study, by Remolina et al. (2012), performed whole‐genome sequencing of a shorter, 50‐generation‐long selection experiment for longevity. Importantly, both Rose (1984) and Remolina et al. (2012) selected for increased lifespan by postponing reproduction, using a design that is qualitatively identical to ours.

**Figure 2:**
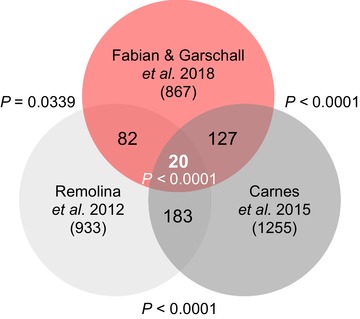
**Sharing of candidate genes across three independent genomic analyses of longevity selection in *Drosophila***. The Venn diagram shows statistically significant overlaps between the candidate genes identified in our study and those of Carnes et al. (2015) and Remolina et al. (2012), calculated with the R package *SuperExactTest* (see Supplementary methods). The results indicate that – across different populations of *D. melanogaster*–there exists genetic parallelism (“gene reusage”) underlying the evolution of longevity. See Table S2 for functional annotations of the shared longevity candidate genes; see Table S5 for statistical details.

We discovered statistically significant sharing of candidate loci across all possible overlaps among the three datasets (Fig. 2, Table S2), indicating genetic parallelism underlying the evolution of late‐life performance. Our dataset contained 147 (11.7%) of the candidate genes of Carnes et al. (2015) and 102 (10.9%) of those of Remolina et al. (2012). Twenty candidate genes (∼2%) were shared across all three studies, representing clear cases of gene reuse during the evolution of longevity and late‐life fertility (Fig. 2, Table S2). Thus, as might be expected from a highly complex and polygenic trait such as lifespan (McElwee et al. 2007), most candidate loci tend to be population‐specific. However, a small but significant proportion of candidate loci is shared among independent populations, perhaps suggesting the existence of “preferred” loci of evolutionary change (Stern and Orgogozo 2008) for longevity. Several of these “high confidence” genes represent promising candidate loci for future functional experiments.

Notably, although each study identified several loci that belong to “canonical” longevity pathways (Guarente and Kenyon 2000; Partridge and Gems 2002; Tatar et al. 2003; Guarente et al. 2008; Kenyon 2010), for example the IIS pathway, the candidate lists and overlaps contain few “classical” lifespan genes that have previously been identified in studies of large‐effect mutants and transgenes. This might be due to a lack of standing variation at these “canonical” longevity loci: perhaps these conserved‐effect loci have been optimized by selection but are now subject to strong purifying selection (see Remolina et al. 2012; Flatt and Partridge 2018). Thus, while segregating IIS polymorphisms with major effects on life‐history traits including lifespan have been identified (Geiger‐Thornsberry and Mackay 2004; Paaby et al. 2010, 2014; Flachsbart et al. 2017; Joshi et al. 2017), our results are consistent with the hypothesis that loci in these canonical pathways might be under selective constraints (see Remolina et al. 2012; Flatt and Partridge 2018).

Even though “canonical” longevity loci seem to be underrepresented, many of the overlapping candidate genes that we have identified have strong empirical support from functional genetics, GWAS, QTL, or gene expression studies, with known roles in lifespan determination, somatic maintenance (e.g., resistance against starvation or oxidative stress, immunity, metabolism), and age‐specific fecundity (see functional annotations in Table S2). The fact that several candidate loci are known to affect age‐specific fecundity is consistent with the age‐at‐reproduction selection regime used by all three studies and possibly also with genetic trade‐offs between early fecundity and lifespan (and/or late‐life fecundity) seen in these selection experiments.

### LONGEVITY CANDIDATE GENES ARE ENRICHED FOR IMMUNE FUNCTION

**Figure 3:**
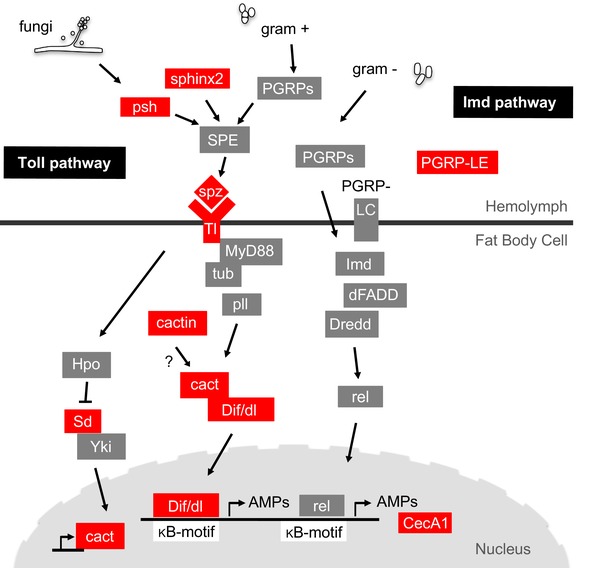
**Genes of the Toll and Imd pathways represent longevity candidates**. Overview of the Toll and Imd pathways, the two major pathways regulating the humoral innate immune response against fungi and gram‐positive bacteria (Toll) and gram‐negative bacteria (Imd). Among our longevity candidates we found an enrichment of immunity‐related genes (enrichment of GO terms associated with “antifungal peptides”). Longevity candidate genes identified in the Toll and Imd pathways are shown in red. For additional immunity‐related candidate genes see Table S4.

We next sought to characterize the functions of our candidate loci with gene ontology (GO) analysis (Kofler and Schlötterer 2012) (Table S3; considering the ontologies “Biological Function,” “Molecular Function,” and ”Cellular Component”). Interestingly, we found an enrichment of candidate genes associated with “antifungal peptides” with a false discovery rate of ∼9% (FDR = 0.085), whereas the term “determination of adult lifespan” had no support (FDR = 1) (Table S3). Immunity against fungi (and gram‐positive bacteria) is regulated by Toll signaling (Belvin and Anderson 1996; Lemaitre et al. 1996; De Gregorio et al. 2002; Valanne et al. 2011), and among our candidates we identified several prominent members of this pathway, including the Toll ligand *spätzle* (*spz*), the receptor *Toll* (*Tl*), the Toll inhibitor *cactus* (*cact*), the NFκB transcription factors *Dorsal‐related immunity factor* (*Dif*) and *dorsal* (*dl*), the upstream serine proteases *persephone* (*psh*) and *sphinx2*, and two regulators of *cactus*, *scalloped* (*sd*) and *cactin* (Fig. 3, Table S4). The other major immune pathway, the Imd pathway (De Gregorio et al. 2002; Kleino and Silverman 2014; Myllymäki et al. 2014), also harbored several but fewer candidates, including *peptidoglycan recognition protein LE* (*PRGP‐LE*) and the antimicrobial peptide *Cecropin A1* (*CecA1*) (Fig. 3, Table S4).

The enrichment of immunity genes prompted us to hypothesize that genetic changes in immune function might contribute to the evolution of longevity and correlated fitness traits (DeVeale et al. 2004; Finch 2007). Importantly, Remolina et al. (2012) also found enrichment of genes involved in “defense response to fungus,” and Carnes et al. (2015) observed divergence in immune gene expression between long‐lived selection and control lines, suggesting that the relation between immunity and lifespan might be general (DeVeale et al. 2004; Finch 2007). While we found a larger number of genes in the Toll pathway, Carnes et al. (2015) and Remolina et al. (2012) found more candidates in the Imd pathway. However, several immune genes are shared across the three studies, despite a relatively small overlap at the individual gene level (Table S4). Immunity might thus represent a general mechanism underlying longevity, with immune genes having pleiotropic effects on lifespan and correlated fitness components.

Despite this compelling commonality across independent experiments, still little is known about how immunity proximately affects longevity and correlated fitness traits; similarly, whether genetic changes in immunity might contribute to the evolution of longer life remains unknown (Garsin et al. 2003; DeVeale et al. 2004; Kurz and Tan 2004; Libert et al. 2006; Troemel et al. 2006; Libert et al. 2008; Fernando et al. 2014; Guo et al. 2014; McCormack et al. 2016; Kounatidis et al. 2017; Loch et al. 2017; Yunger et al. 2017). We therefore aimed to test whether the evolved genomic signature of immune gene enrichment observed in our study – and similarly by Carnes et al. (2015) and Remolina et al. (2012)–might represent a physiological mechanism underlying evolutionary changes in lifespan and late‐life fertility.

### LONG‐LIVED FLIES SHOW REDUCED IMMUNE INDUCTION WITH AGE

We first examined whether the selection and control lines differ in the expression of antimicrobial peptides (AMPs), the major effectors of the innate immune response. We used three AMPs as readouts of Toll and Imd signaling activity, *Drosomycin* (*Drs*), *Attacin A* (*AttA*), and *Diptericin* (*Dpt*). *Drs* and *AttA* are regulated by both Toll and Imd signaling, whereas *Dpt* is mainly regulated by the Imd pathway (De Gregorio et al. 2002). Using quantitative real‐time PCR, we determined mRNA levels of young (5–6‐day‐old) and aged (25–26‐day‐old) female flies, either without pricking, upon aseptic pricking (mock control) or upon prick infection with *Erwinia carotovora carotovora 15* (*Ecc 15*). Systemic infections with this bacterium induce the expression of all three AMPs assayed here (Lemaitre et al. 1997; Basset et al. 2000; De Gregorio et al. 2002).

**Figure 4:**
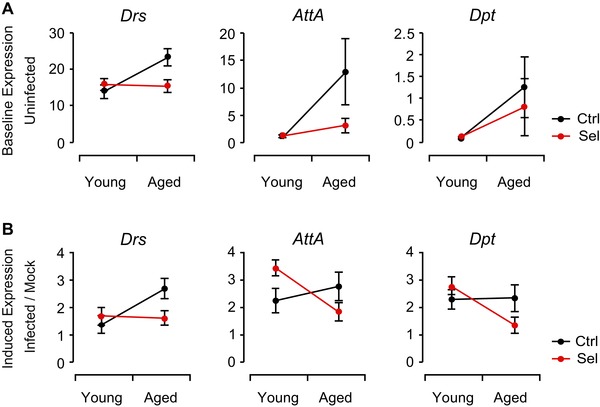
**Age‐dependent differential expression of immunity genes. (A)** Baseline mRNA expression levels of three antimicrobial peptides (AMPs), *Drosomycin* (*Drs*), *Attacin A* (*AttA*), and *Diptericin* (*Dpt*) in uninfected (nonpricked) young (5–6‐day‐old) and aged (25–26‐day‐old) female flies. The panel shows relative expression levels (based on efficiency‐corrected ∆Ct‐values), normalized to the geometric mean of two control transcripts, *Rp49* (*RpL32*) and *Gapdh2*. Unselected control flies upregulate AMP expression with age, but selected flies do not (ANOVA; significant Age x Regime interactions for *Drs*: *P* = 0.003 and for *AttA*: *P* = 0.005; while for *Dpt* the interaction was not significant, a post‐hoc test revealed that at old age *Dpt* levels were significantly lower in selected than in control flies: *P* = 0.038). Error bars shows standard errors of the mean. See Table S5 for full details of statistical analysis. **(B)** Induction of *Drs*, *AttA*, and *Dpt* upon prick infection of young (5–6‐day‐old) and aged (25–26‐day‐old) female flies with *Erwinia carotovora carotovora 15* (*Ecc15*) relative to aseptic prick (mock) controls, 4–6 hours after jabbing. The panel shows the ratio of the expression values for infected relative to uninfected (mock prick control) flies, based on efficiency‐corrected ∆Ct‐values normalized to the geometric mean of two control transcripts, *Rp49* (*RpL32*) and *Gapdh2*. Relative to mock infected flies, AMP induction upon infection in long‐lived flies tends to be slightly higher at young age, but lower at old age (ANOVA on expression ratios (infected/mock infected); significant Age x Regime interactions for *Drs*: *P* = 0.026 and for *AttA*: *P* = 0.03; the same trend, albeit not significant, is seen for *Dpt*). Error bars shows standard errors of the mean. Full statistical details are given in Table S5.

Without pricking, control flies upregulated AMP baseline expression with age (Fig. 4A) – a pattern that is commonly observed in wild‐type flies and attributed to persistent chronic infection and a prolonged immune response at old age (Seroude et al. 2002; DeVeale et al. 2004; Zerofsky et al. 2005; Ren et al. 2007; Ramsden et al. 2008). In marked contrast to control flies, baseline AMP levels remained constant as a function of age in selected flies (Fig. 4A).

AMP expression also differed substantially between control and selected flies upon infection: at young age, the AMP response was slightly stronger in long‐lived flies than in control flies, whereas at old age long‐lived flies tended to downregulate AMP induction (Fig. 4b). Thus, unlike aged wild‐type flies which upregulate AMPs but suffer from immunosenescence and show signs consistent with chronic inflammation (i.e., reduced infection survival, increased bacterial load, more persistent AMP induction upon infection; see Zerofsky et al. 2005; Ren et al. 2007; Ramsden et al. 2008; Myllymäki et al. 2014), aged long‐lived selected flies exhibit restrained AMP expression.

Our results therefore suggest that long‐lived flies might have evolved improved age‐dependent immune homeostasis and alleviated immunosenescence (DeVeale et al. 2004). These evolutionary changes in immune gene induction might also be linked to the late‐life fertility of the long‐lived lines. Since in our selection experiment lifespan was selected for by postponing reproduction, the observed differences in immune gene induction between the regimes might be a byproduct of selection for increased late‐life fertility in the long‐lived selection lines. This would be consistent with the observation that infection reduces fecundity: infection‐induced synthesis of AMPs incurs a cost of reproduction in wild‐type flies but this cost is abolished in Imd pathway mutants (Zerofsky et al. 2005).

### LONG‐LIVED FLIES HAVE IMPROVED SURVIVAL UPON INFECTION

**Figure 5:**
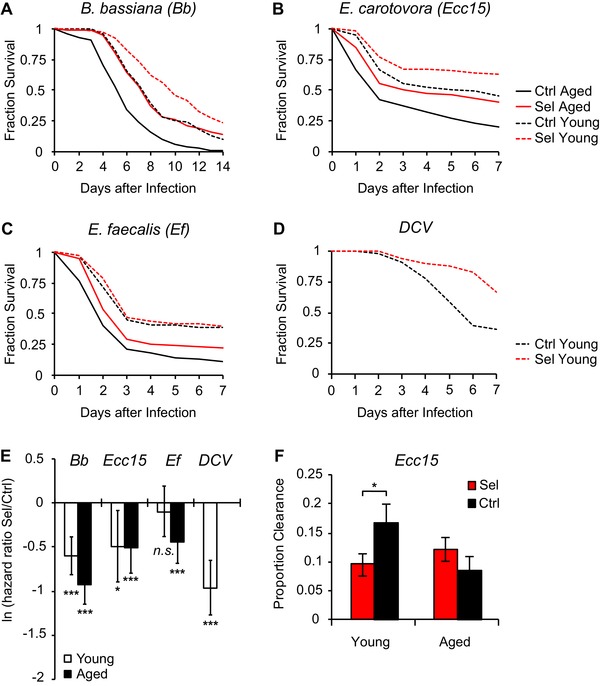
**Long‐lived flies survive infections better than control flies. (A–D)** Survival of selected and control flies upon infection with the fungus *Beauveria bassiana* (*Bb*) **(A)**, the gram‐negative bacterium *Erwinia carotovora carotovora 15* (*Ecc15*) **(B)**, the gram‐positive bacterium *Enterococcus faecalis* (*Ef*) **(*C*)**, and with *Drosophila* C virus (*DCV*) **(D)**. Except for DCV infection, assays were performed with both young (1–4‐days‐old) and aged (22–25‐days‐old) female flies. All survival assays were terminated after 7 days and the remaining flies censored for analysis. Red curves show average survival of selection lines and black curves survival of control lines; dashed lines represent young flies and solid lines aged flies. For statistics see Fig. 4E and Table S5. **(E)** Summary of infection‐induced mortality in selection and control lines. Shown are estimates of the hazard ratios of selection relative to control lines; negative values indicate superior survival of selection lines relative to control lines. *P*‐values for the effect of regime are from Cox (proportional hazards) regression with *χ* ^2^ tests; ^*^ *P* < 0.05, ^***^ *P* < 0.001. Error bars show the lower and upper 95^th^ percentiles; see Table S5 for statistical details. **(F)** Clearance ability of selection and control lines over a 6‐day postinfection period. Percentage of successful (100%) clearance of young (5–6‐days old) and aged (23–25‐days old) female flies after infection with *Ecc15*. Error bars show binomial standard errors. Binomial GLM revealed a significant Age x Regime interaction (*P* = 0.018): clearance stays constant with age in selected flies but starts out higher and then declines with age in control flies; this might be consistent with the hypothesis that selected flies are more tolerant. Details of statistical analysis are given in Table S5.

To investigate whether selected and control flies differ in realized immune function we measured their survival after infection with four different pathogens (Fig. 5). Long‐lived flies survived infections with a fungus (*Beauveria bassiana*, *Bb*), with the Gram‐negative bacterium *Ecc15* and with the Gram‐positive bacterium *Enterococcus faecalis* (*Ef*) overall markedly better than control flies (Fig. 5A,B,C,E). Improved survival of long‐lived flies was observed for both young and aged flies after infection with *Bb* and *Ecc15*, whereas for *Ef* infection only aged long‐lived flies showed increased survival relative to controls (Fig. 5A,B,C,E). Because one of our candidate genes, the JAK/STAT activating cytokine *unpaired3* (*upd3*; Table S4), is involved in antiviral immunity (Zhu et al. 2013), we also measured the survival of flies upon infection with *Drosophila* C virus (DCV). This assay was carried out only with young, not aged flies, but we again found that long‐lived flies survived infection with DCV much better than control flies (Fig. 5D,E). The evolution of prolonged lifespan might thus be accompanied–or partly be caused – by selection for improved realized immunity.

Next, we examined the ability of selection and control flies to successfully clear bacterial (*Ecc15*) infections over a 6‐day period postinfection. The ability of control flies to clear an infection was higher than that of long‐lived flies at young age but declined at old age; in contrast, clearance was overall lower in long‐lived flies yet did not change with age (Fig. 5F). The lower clearance ability of long‐lived selected flies, independent of their age, together with their improved survival upon infection, possibly indicates that they have evolved to be more tolerant to infections than unselected control flies (Best et al. 2008; Ayres and Schneider 2008, 2012; Felix et al. 2012).

### REDUCED TOLL SIGNALING EXTENDS LIFESPAN BUT OVERACTIVATION IS DETRIMENTAL

Our results above support the idea that improved age‐dependent regulation of immunity contributes to longevity and late‐life fertility, but how immune genes affect lifespan is not well studied, especially in *Drosophila* (DeVeale et al. 2004; Libert et al. 2006; Fernando et al. 2014; Guo et al. 2014; Kounatidis et al. 2017; Loch et al. 2017). For example, previous work has shown that constitutive upregulation of the peptidoglycan recognition proteins PGRP‐LE and PGRP‐LC causes hyperactivation of Imd signaling and reduces lifespan (DeVeale et al. 2004; Libert et al. 2006). Similarly, several mutants of negative regulators of Imd signaling display shortened lifespan (Fernando et al. 2014; Kounatidis et al. 2017). While we also identified *PGRP‐LE* as a lifespan candidate gene, most immunity genes in our analysis belong to the Toll pathway (Fig. 3, Table S4).

To examine whether Toll signaling affects lifespan, we used transgenic RNAi to silence four longevity candidate genes of the Toll pathway: the ligand *spz*, the receptor *Tl*, the inhibitor *cact*, and the NFκB transcription factor *Dif*. To prevent deleterious side effects of knocking down these developmentally critical genes (Nüsslein‐Volhard and Wieschaus 1980; Belvin and Anderson 1996) we used a mifepristone‐inducible *daughterless* (*da*)‐GeneSwitch(GS)‐GAL4 driver (Tricoire et al. 2009) to direct expression of UAS‐RNAi constructs against these genes specifically during adulthood and throughout the fly body.

**Figure 6:**
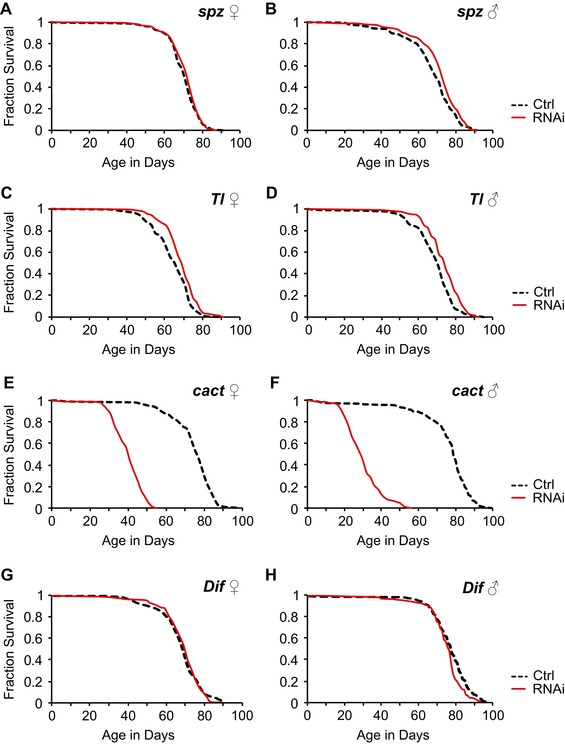
**Decreased Toll signaling promotes longevity while hyperactivation shortens lifespan. (**A–H) Adult survival upon ubiquitous, adult‐specific transgenic RNAi directed against four canonical components of the Toll signaling pathway: the Toll ligand *spätzle* (*spz*) **(A, B)**, the receptor *Toll* (*Tl*) **(C, D)**, the Toll inhibitor *cactus* (*cact*) **(E, F)**, and the NFκB transcription factor *Dorsal‐related immunity factor* (*Dif*) **(G, H)**. **(A, C, E, G)** show data for female flies and **(B, D, F, H)** represent data for male flies. Silencing the *Tl* receptor **(C, D)** – but not the *spz* ligand **(A, B)**–extends lifespan, while silencing the antagonist *cact* dramatically shortens lifespan **(E, F)**; silencing *Dif* has opposite effects on female and male lifespan **(G, H)**. For details of statistical analysis using mixed‐effects Cox (proportional hazards) regression see Table S5. Expression of the different UAS‐RNAi responder constructs was driven with a mifepristone‐inducible *daughterless*(*da*)‐GeneSwitch(GS)‐GAL4 driver. Solid red curves: 200 μg/mL (466 μM) mifepristone (RNAi); dashed curves: 0 μg/mL mifepristone (control). For experimental details see Supplementary methods.

Downregulation of the *Tl* receptor–but not of its ligand *spz*–mildly but significantly extended lifespan (Fig. 6A,B,C,D), while silencing the antagonist *cact*–leading to Toll pathway hyperactivation (Lemaitre et al. 1996; Aggarwal and Silverman 2008)–drastically reduced lifespan (Fig. 6E,F), similar to the effects of overactivation or derepression of Imd signaling (DeVeale et al. 2004; Libert et al. 2006; Guo et al. 2014; Kounatidis et al. 2017). Interestingly, we found opposite lifespan effects of *Dif*‐RNAi for females (Fig. 6G) and males (Fig. 6H). In agreement with our findings for females, two studies have previously found that a loss‐of‐function mutant of *Dif* lives longer than wild‐type (Le Bourg et al. 2012; Petersen et al. 2013), but why silencing *Dif* reduces male lifespan remains unclear. Our results thus establish that downregulation of Toll signaling increases lifespan (albeit weakly so), whereas overactivation of this pathway strongly shortens life.

Our findings for the Toll pathway are also consistent with recent studies of IMD signaling showing that lifespan is extended under conditions of reduced lifetime IMD activity (Loch et al. 2017) or when the IMD AMPs *AttacinC* (*AttC*) and *Diptericin B* (*DiptB*) are downregulated in the fat body (Lin et al. 2018). The evidence available to date therefore suggests that decreased activity of the immune system can promote lifespan (DeVeale 2004), possibly by reducing the costs of immune deployment (McKean and Lazzaro 2011). Moreover, as we show here, longer lifespan can evolve – at least partly – via evolutionary changes in immunity.

## Conclusion

Explaining the genetic basis of variation in longevity is a longstanding problem in evolutionary genetics and the biology of aging (Finch 1990; Rose 1991; Zwaan 1999; Partridge and Gems 2006; Flatt and Schmidt 2009; Flatt and Partridge 2018). Here we have performed a whole‐genome sequencing analysis of an over 35‐year‐long selection experiment for postponed aging and late‐life fertility in *Drosophila* (Luckinbill et al. 1984).

Notably, among the longevity candidate genes identified in our genomic screen, we found an enrichment of immune genes, especially in the Toll pathway. By comparing our data to those from two previous genomic studies of longevity selection in *Drosophila* (Remolina et al. 2012; Carnes et al. 2015) we infer that–while different studies might identify different immune genes as longevity candidates–immune function likely represents a general process‐level mechanism underlying the evolution of longevity assurance and of late‐life performance (Martin et al. 1996; Partridge and Gems 2002; McElwee et al. 2007). This is particularly noteworthy in view of the growing evidence that aging, inflammation and immunity are intricately linked at the molecular level (DeVeale et al. 2004 ; Kurz and Tan 2004; Finch 2007; Salminen et al. 2008; Eleftherianos and Castillo 2012). However, how immunity contributes to longevity and correlated fitness traits is largely unclear.

While aged wild‐type flies upregulate immune gene expression (Pletcher et al. 2002; Seroude et al. 2002; Landis et al. 2004), they typically have a reduced capacity to fight off and survive infections, suggesting that they suffer from immune overactivation and immunopathology (Zerofsky et al. 2005; Ren et al. 2007; Ramsden et al. 2008). Here, we show that long‐lived flies, by contrast, tend to downregulate the induction of immune effector genes (AMPs) with age yet have substantially improved survivorship upon infection. This seems to confirm that elevated immune gene expression at old age might either be ineffective or even detrimental, perhaps as a consequence of senescent dysregulation of gene expression (Zerofsky et al. 2005; Khan et al. 2017). The downregulation of AMPs seen in the long‐lived selection lines might also be a byproduct of selection for late‐life fertility in these lines since elevated AMP expression upon infection is known to reduce fecundity (Zerofsky et al. 2005).

Since optimal immunity depends on the balance between efficient clearance of pathogens and limiting immunity‐induced damage (Cassedevall and Pirofski 1999; Read et al. 2008; Råberg et al. 2009; Medzhitov et al. 2012), we propose that selection for longevity and late‐life fertility leads to improved age‐dependent immune homeostasis and alleviates the trade‐off between immunity and immunopathology. This trade‐off can be decoupled to some degree by tolerance mechanisms (Medzhitov et al. 2012), suggesting that the improved immunity of long‐lived flies might–at least in part–be due to increased tolerance. In line with the notion of a trade‐off between immunity and immunity‐induced damage, work in the mealworm beetle shows that deployment of the immune effector phenoloxidase (PO) causes early‐life inflammation, faster aging, and immunopathology at old age, whereas RNAi silencing of PO extends lifespan and improves survival after infection (Khan et al. 2017). This is consistent with the fact that hyperactivation or derepression of Imd signaling (DeVeale et al. 2004; Libert et al. 2006; Fernando et al. 2014; Kounatidis et al. 2017) and, as we observe here, of Toll signaling reduces lifespan. Conversely, we find that adult downregulation of Toll signaling mildly promotes lifespan, similar to recent findings for the Imd pathway (Kounatidis et al. 2017; Lin et al. 2018).

Together, our work reveals the existence of a causal–but mechanistically still poorly understood–link between improved age‐dependent immunity and the evolution of longevity and late‐life fertility (Garschall and Flatt 2018). This relationship clearly warrants further mechanistic and evolutionary study.

## Methods

All methods are given in the Supplementary methods file (see Supporting Information section below), including details of selection and control lines, next‐generation sequencing, bioinformatic, and statistical analyses, gene expression analyses, immunity assays, transgenic RNAi, and lifespan assays.

## CONFLICT OF INTEREST

The authors declare no conflict of interest.

Associate Editor: Rhonda Snook

## Supporting information


**Supplementary methods** (pdf). Description of all methods, including details of selection and control lines, next‐generation sequencing, bioinformatic and statistical analyses, gene expression analyses, immunity assays, transgenic RNAi and lifespan assays.

**Table S1**. (xls). Longevity candidate SNPs and candidate genes.

**Table S2**. (xls). Shared candidate genes across three independent studies.

**Table S3**. (xls). Gene ontology (GO) analysis of longevity candidate genes.

**Table S4**. (xls). Immunity genes implicated in lifespan and aging.

**Table S5**. (xls). Full statistical details of data analyses shown in the main text.

## References

[evl389-bib-0001] Aggarwal, K. , and N. Silverman . 2008 Positive and negative regulation of the *Drosophila* immune response. BMB Rep. 41:267–277. 18452646 10.5483/bmbrep.2008.41.4.267

[evl389-bib-0002] Akey, J. M. 2009 Constructing genomic maps of positive selection in humans: where do we go from here? Genome Res. 19:711–722. 19411596 10.1101/gr.086652.108 PMC3647533

[evl389-bib-0003] Arking, R. 1987 Successful selection for increased longevity in *Drosophila*: analysis of the survival data and presentation of a hypothesis on the genetic regulation of longevity. Exp. Gerontol. 22:199–220. 3113991 10.1016/0531-5565(87)90040-4

[evl389-bib-0004] Ayres, J. S. , and D. S. Schneider . 2008 A signaling protease required for melanization in *Drosophila* affects resistance and tolerance of infections. PLoS Biol. 6:e305. 10.1371/journal.pbio.0060305 PMC2596860 19071960

[evl389-bib-0005] Ayres, J. S. , and D. S. Schneider . 2012 Tolerance of infections. Ann. Rev. Immunol. 30:271–294. 22224770 10.1146/annurev-immunol-020711-075030

[evl389-bib-0006] Basset, A. , R. S. Khush , A. Braun , L. Gardan , F. Boccard , J. A. Hoffmann et al. 2000 The phytopathogenic bacteria *Erwinia carotovora* infects *Drosophila* and activates an immune response. Proc. Natl. Acad. Sci. USA 97:3376–3381. 10725405 10.1073/pnas.070357597 PMC16247

[evl389-bib-0007] Belvin, M. P. , and K. V. Anderson . 1996 A conserved signaling pathway: the *Drosophila* toll‐dorsal pathway. Ann. Rev. Cell Dev. Biol. 12:393–416. 8970732 10.1146/annurev.cellbio.12.1.393

[evl389-bib-0008] Best, A. , A. White , and M. Boots . 2008 Maintenance of host variation in tolerance to pathogens and parasites. Proc. Natl. Acad. Sci. USA 105:20786–20791. 19088200 10.1073/pnas.0809558105 PMC2634923

[evl389-bib-0009] Carbone, M. A. , K. W. Jordan , R. F. Lyman , S. T. Harbison , J. Leips , T. J. Morgan et al. 2006 Phenotypic variation and natural selection at Catsup, a pleiotropic quantitative trait gene in *Drosophila* . Curr. Biol. 16:912–919. 16682353 10.1016/j.cub.2006.03.051 PMC10766118

[evl389-bib-0010] Carnes, M. U. , T. Campbell , W. Huang , D. G. Butler , M. A. Carbone , L. H. Duncan et al. 2015 The genomic basis of postponed senescence in *Drosophila melanogaster* . PLoS One 10:e0138569. 26378456 10.1371/journal.pone.0138569 PMC4574564

[evl389-bib-0011] Casadevall, A. , and L. A. Pirofski . 1999 Host‐pathogen interactions: redefining the basic concepts of virulence and pathogenicity. Infect. Immun. 67:3703–3713. 10417127 10.1128/iai.67.8.3703-3713.1999 PMC96643

[evl389-bib-0012] Conte, G. L. , M. E. Arnegard , C. L. Peichel , and D. Schluter . 2012 The probability of genetic parallelism and convergence in natural populations. Proc. Roy. Soc. B 279:5039–5047. 10.1098/rspb.2012.2146 PMC3497250 23075840

[evl389-bib-0013] De Gregorio, E. , P. T. Spellman , P. Tzou , G. M. Rubin , and B. Lemaitre . 2002 The Toll and Imd pathways are the major regulators of the immune response in *Drosophila* . EMBO J. 21:2568–2579. 12032070 10.1093/emboj/21.11.2568 PMC126042

[evl389-bib-0014] De Luca, M. , N. V. Roshina , G. L. Geiger‐Thornsberry , R. F. Lyman , E. G. Pasyukova , and T. F. Mackay . 2003 Dopa decarboxylase (Ddc) affects variation in *Drosophila* longevity. Nat. Genet. 34:429–433. 12881721 10.1038/ng1218

[evl389-bib-0015] DeVeale, B. , T. Brummel , and L. Seroude . 2004 Immunity and aging: the enemy within? Aging Cell 3:195–208. 15268753 10.1111/j.1474-9728.2004.00106.x

[evl389-bib-0016] Eleftherianos, I. , and J. C. Castillo . 2012 Molecular mechanisms of aging and immune system regulation in *Drosophila* . Int. J. Mol. Sci. 13:9826–9844. 22949833 10.3390/ijms13089826 PMC3431831

[evl389-bib-0017] Felix, T. M. , K. A. Hughes , E. A. Stone , J. M. Drnevich , and J. Leips . 2012 Age‐specific variation in immune response in *Drosophila melanogaster* has a genetic basis. Genetics 191:989–1002. 22554890 10.1534/genetics.112.140640 PMC3389989

[evl389-bib-0018] Fernando, M. D. A. , I. Kounatidis , and P. Ligoxygakis . 2014 Loss of trabid, a new negative regulator of the *Drosophila* immune‐deficiency pathway at the level of TAK1, reduces life span. PLoS Genet. 10:e1004117. 24586180 10.1371/journal.pgen.1004117 PMC3930493

[evl389-bib-0019] Finch, C. E. 1990 Longevity, senescence, and the genome. Chicago Univ. Press, Chicago.

[evl389-bib-0020] Finch, C. E. 2007 The biology of human longevity—Inflammation, nutrition, and aging in the evolution of lifespans. Academic Press, San Diego.

[evl389-bib-0021] Flachsbart, F. , J. Dose , L. Gentschew , C. Geismann , A. Caliebe , C. Knecht et al. 2017 Identification and characterization of two functional variants in the human longevity gene FOXO3. Nat. Comm. 8:2063. 10.1038/s41467-017-02183-y PMC5727304 29234056

[evl389-bib-0022] Flatt T., and Heyland A., eds. 2011 Mechanisms of life history evolution The genetics and physiology of life history traits and trade‐offs. Oxford Univ. Press, Oxford.

[evl389-bib-0023] Flatt, T. , and L. Partridge . 2018 Horizons in the evolution of aging. BMC Biol. 16:93. 30124168 10.1186/s12915-018-0562-z PMC6100731

[evl389-bib-0024] Flatt, T. , and P. S. Schmidt . 2009 Integrating evolutionary and molecular genetics of aging. Biochim. Biophys. Acta 1790:951–962. 19619612 10.1016/j.bbagen.2009.07.010 PMC2972575

[evl389-bib-0025] Garschall, K. , and T. Flatt . 2018 The interplay between immunity and aging in *Drosophila* . F1000Research 2018, 7(F1000 Faculty Rev):160. 10.12688/f1000research.13117.1 PMC5806056 29487742

[evl389-bib-0026] Garsin, D. A. , J. M. Villanueva , J. Begun , D. H. Kim , C. D. Sifri , S. B. Calderwood et al. 2003 Long‐lived *C. elegans* daf‐2 mutants are resistant to bacterial pathogens. Science 300:1921. 12817143 10.1126/science.1080147

[evl389-bib-0027] Geiger‐Thornsberry, G. L. , and T. F. Mackay . 2004 Quantitative trait loci affecting natural variation in *Drosophila* longevity. Mech. Ageing. Dev. 125:179–189. 15013662 10.1016/j.mad.2003.12.008

[evl389-bib-0028] Guarente, L. , and C. Kenyon . 2000 Genetic pathways that regulate ageing in model organisms. Nature 408:255–262. 11089983 10.1038/35041700

[evl389-bib-0029] Guarente L. P., Partridge L., and Wallace D. C., eds. 2008 Molecular biology of aging. Cold Spring Harbor Laboratory Press, New York.

[evl389-bib-0030] Guo, L. , J. Karpac , S. L. Tran , and H. Jasper . 2014 PGRP‐SC2 promotes gut immune homeostasis to limit commensal dysbiosis and extend lifespan. Cell 156:109–122. 24439372 10.1016/j.cell.2013.12.018 PMC3928474

[evl389-bib-0031] Joshi, P. K. , N. Pirastu , K. A. Kentistou , K. Fischer , E. Hofer et al. 2017 Genome‐wide meta‐analysis associates HLA‐DQA1/DRB1 and LPA and lifestyle factors with human longevity. Nat. Comm. 8:910. 10.1038/s41467-017-00934-5 PMC5715013 29030599

[evl389-bib-0032] Kenyon, C. J. 2010 The genetics of ageing. Nature 464:504–512. 20336132 10.1038/nature08980

[evl389-bib-0033] Khan, I. , D. Agashe , and J. Rolff . 2017 Early‐life inflammation, immune response and ageing. Proc. Roy. Soc. B 284:20170125. 10.1098/rspb.2017.0125 PMC5360934 28275145

[evl389-bib-0034] Kleino, A. , and N. Silverman . 2014 The *Drosophila* IMD pathway in the activation of the humoral immune response. Dev. Comp. Immun. 42:25–35. 10.1016/j.dci.2013.05.014 PMC3808521 23721820

[evl389-bib-0035] Kofler, R. , and C. Schlötterer . 2012 Gowinda: unbiased analysis of gene set enrichment for genome‐wide association studies. Bioinformatics 28:2084–2085. 22635606 10.1093/bioinformatics/bts315 PMC3400962

[evl389-bib-0036] Kounatidis, I. , S. Chtarbanova , Y. Cao , M. Hayne , D. Jayanth , B. Ganetzky et al. 2017 NF‐kB immunity in the brain determines fly lifespan in healthy aging and age‐related neurodegeneration. Cell Rep. 19:836–848. 28445733 10.1016/j.celrep.2017.04.007 PMC5413584

[evl389-bib-0037] Kurz, C. L. , and M. W. Tan . 2004 Regulation of aging and innate immunity in *C. elegans* . Aging Cell 3:185–193. 15268752 10.1111/j.1474-9728.2004.00108.x

[evl389-bib-0038] Landis, G. N. , D. Abdueva , D. Skvortsov , J. Yang , B. E. Rabin , J. Carrick et al. 2004 Similar gene expression patterns characterize aging and oxidative stress in *Drosophila melanogaster* . Proc. Natl. Acad. Sci. USA 101:7663–7668. 15136717 10.1073/pnas.0307605101 PMC419663

[evl389-bib-0039] Le Bourg, E. , K. Malod , and I. Massou . 2012 The NF‐kB‐like factor DIF could explain some positive effects of a mild stress on longevity, behavioral aging, and resistance to strong stresses in *Drosophila melanogaster* . Biogerontology 13:445–455. 22791143 10.1007/s10522-012-9389-0

[evl389-bib-0040] Lemaitre, B. , E. Nicolas , L. Michaut , J. M. Reichhart , and J. A. Hoffmann . 1996 The dorsoventral regulatory gene cassette spatzle/Toll/cactus controls the potent antifungal response in *Drosophila* adults. Cell 86:973–983. 8808632 10.1016/s0092-8674(00)80172-5

[evl389-bib-0041] Lemaitre, B. , J.‐M. Reichhart , and J. A. Hoffmann . 1997 *Drosophila* host defense: differential induction of antimicrobial peptide genes after infection by various classes of microorganisms. Proc. Natl. Acad. Sci. USA 94:14614–14619. 9405661 10.1073/pnas.94.26.14614 PMC25070

[evl389-bib-0042] Lewontin, R. C. , and J. Krakauer . 1973 Distribution of gene frequency as a test of the theory of the selective neutrality of polymorphisms. Genetics 74:175–195. 4711903 10.1093/genetics/74.1.175 PMC1212935

[evl389-bib-0043] Libert, S. , Y. Chao , X. Chu , and S. D. Pletcher . 2006 Trade‐offs between longevity and pathogen resistance in *Drosophila melanogaster* are mediated by NFkappaB signaling. Aging Cell 5:533–543. 17129215 10.1111/j.1474-9726.2006.00251.x

[evl389-bib-0044] Libert, S. , Y. Chao , J. Zwiener , and S. D. Pletcher . 2008 Realized immune response is enhanced in long‐lived puc and chico mutants but is unaffected by dietary restriction. Mol. Immun. 45:810–817. 10.1016/j.molimm.2007.06.353 17681604

[evl389-bib-0045] Lin, Y. R. , H. Parikh , and Y. Park . 2018 Stress resistance and lifespan enhanced by downregulation of antimicrobial peptide genes in the Imd pathway. Aging 10:622–631. 29677000 10.18632/aging.101417 PMC5940113

[evl389-bib-0046] Loch, G. , I. Zinke , T. Mori , P. Carrera , J. Schroer , H. Takeyama et al. 2017 Antimicrobial peptides extend lifespan in *Drosophila* . PLoS ONE 12:e0176689. 28520752 10.1371/journal.pone.0176689 PMC5435158

[evl389-bib-0047] Luckinbill, L. , and M. Clare . 1985 Selection for life span in *Drosophila melanogaster* . Heredity 55:9–18. 3930429 10.1038/hdy.1985.66

[evl389-bib-0048] Luckinbill, L. S. , R. Arking , M. J. Clare , W. C. Cirocco , and S. A. Buck . 1984 Selection for delayed senescence in *Drosophila melanogaster* . Evolution 38:996–1003. 28555795 10.1111/j.1558-5646.1984.tb00369.x

[evl389-bib-0049] Martin, G. M. , S. N. Austad , and T. E. Johnson . 1996 Genetic analysis of ageing: role of oxidative damage and environmental stresses. Nat. Genet. 13:25–34. 8673100 10.1038/ng0596-25

[evl389-bib-0050] McCormack, S. , S. Yadav , U. Shokal , E. Kenney , D. Cooper , and I. Eleftherianos . 2016 The insulin receptor substrate Chico regulates antibacterial immune function in *Drosophila* . Immun. Age. 13:15. 10.1186/s12979-016-0072-1 PMC4852101 27134635

[evl389-bib-0051] McElwee, J. J. , E. Schuster , E. Blanc , M. D. Piper , J. H. Thomas , D. S. Patel et al. 2007 Evolutionary conservation of regulated longevity assurance mechanisms. Genome Biol. 8:R132. 17612391 10.1186/gb-2007-8-7-r132 PMC2323215

[evl389-bib-0052] McKean, K. A. , and B. P. Lazzaro . 2011 The costs of immunity and the evolution of immunological defense mechanisms Pp. 299–310 *in* Flatt T. and Heyland A., eds. Mechanisms of life history evolution—The genetics and physiology of life history traits and trade‐offs. Oxford Univ. Press, Oxford.

[evl389-bib-0053] Medzhitov, R. , D. Schneider , and M. P. Soares . 2012 Disease tolerance as a defense strategy. Science 335:936–941. 22363001 10.1126/science.1214935 PMC3564547

[evl389-bib-0054] Myllymäki, H. , S. Valanne , and M. Rämet . 2014 The *Drosophila* Imd signaling pathway. J. Immunol. 192:3455–3462. 24706930 10.4049/jimmunol.1303309

[evl389-bib-0055] Nüsslein‐Volhard, C. , and E. Wieschaus . 1980 Mutations affecting segment number and polarity in *Drosophila* . Nature 287:795–801. 6776413 10.1038/287795a0

[evl389-bib-0056] Paaby, A. B. , M. J. Blacket , A. A. Hoffmann , and P. S. Schmidt . 2010 Identification of a candidate adaptive polymorphism for *Drosophila* life history by parallel independent clines on two continents. Mol. Ecol. 19:760–774. 20074316 10.1111/j.1365-294X.2009.04508.x

[evl389-bib-0057] Paaby, A. B. , A. O. Bergland , E. L. Behrman , and P. S. Schmidt . 2014 A highly pleiotropic amino acid polymorphism in the *Drosophila* insulin receptor contributes to life‐history adaptation. Evolution 68:3395–3409. 25319083 10.1111/evo.12546 PMC5079517

[evl389-bib-0058] Partridge, L. , and D. Gems . 2002 Mechanisms of ageing: public or private? Nat. Rev. Genet. 3:165–175. 11972154 10.1038/nrg753

[evl389-bib-0059] Partridge, L. , and D. Gems . 2006 Beyond the evolutionary theory of ageing, from functional genomics to evo‐gero. Trends Ecol. Evol. 21:334–340. 16769434 10.1016/j.tree.2006.02.008

[evl389-bib-0060] Partridge, L. , N. Prowse , and P. Pignatelli . 1999 Another set of responses and correlated responses to selection on age at reproduction in *Drosophila melanogaster* . Proc. Roy. Soc. London B 266:255–261. 10.1098/rspb.1999.0630 PMC1689678 10081162

[evl389-bib-0061] Pasyukova, E. G. , N. V. Roshina , and T. F. C. Mackay . 2004 Shuttle craft: a candidate quantitative trait gene for *Drosophila* lifespan. Aging Cell 3:297–307. 15379853 10.1111/j.1474-9728.2004.00114.x

[evl389-bib-0062] Petersen, A. J. , R. J. Katzenberger , and D. A. Wassarman . 2013 The innate immune response transcription factor relish is necessary for neurodegeneration in a *Drosophila* model of ataxia‐telangiectasia. Genetics 194:133–142. 23502677 10.1534/genetics.113.150854 PMC3632461

[evl389-bib-0063] Pletcher, S. D. , S. J. Macdonald , R. Marguerie , U. Certa , S. C. Stearns , D. B. Goldstein et al. 2002 Genome‐wide transcript profiles in aging and calorically restricted *Drosophila melanogaster* . Curr. Biol. 12:712–723. 12007414 10.1016/s0960-9822(02)00808-4

[evl389-bib-0064] Ramsden, S. , Y. Y. Cheung , and L. Seroude . 2008 Functional analysis of the *Drosophila* immune response. Aging Cell 7:225–236. 18221416 10.1111/j.1474-9726.2008.00370.x

[evl389-bib-0065] Read, A. F. , A. L. Graham , and L. Råberg . 2008 Animal defenses against infectious agents: is damage control more important than pathogen control. PLoS Biol. 6:e1000004. 10.1371/journal.pbio.1000004 PMC2605932 19222305

[evl389-bib-0066] Remolina, S. C. , P. L. Chang , J. Leips , S. V. Nuzhdin , and K. A. Hughes . 2012 Genomic basis of aging and life history evolution in *Drosophila melanogaster* . Evolution 66:3390–3403. 23106705 10.1111/j.1558-5646.2012.01710.x PMC4539122

[evl389-bib-0067] Ren, C. , P. Webster , S. E. Finkel , and J. Tower . 2007 Increased internal and external bacterial load during *Drosophila* aging without life‐span trade‐off. Cell Metab. 6:144–152. 17681150 10.1016/j.cmet.2007.06.006

[evl389-bib-0068] Rose, M. R. 1984 Laboratory evolution of postponed senescence *in Drosophila melanogaster* . Evolution 38:1004–1010. 28555803 10.1111/j.1558-5646.1984.tb00370.x

[evl389-bib-0069] Rose, M. R. 1991 Evolutionary biology of aging. Oxford Univ. Press, New York.

[evl389-bib-0070] Råberg, L. , A. L. Graham , and A. F. Read . 2009 Decomposing health: tolerance and resistance to parasites in animals. Phil. Trans. Roy. Soc. B 364:37–49. 18926971 10.1098/rstb.2008.0184 PMC2666700

[evl389-bib-0071] Salminen, A. , J. Huuskonen , J. Ojala , A. Kauppinen , K. Kaarniranta , and T. Suuronen . 2008 Activation of innate immunity system during aging: NF‐kB signaling is the molecular culprit of inflamm‐aging. Age. Res. Rev. 7:83–105. 10.1016/j.arr.2007.09.002 17964225

[evl389-bib-0072] Schlötterer, C. , R. Tobler , R. Kofler , and V. Nolte . 2014 Sequencing pools of individuals—mining genome‐wide polymorphism data without big funding. Nat. Rev. Genet. 15:749–763. 25246196 10.1038/nrg3803

[evl389-bib-0073] Seroude, L. , T. Brummel , P. Kapahi , and S. Benzer . 2002 Spatio‐temporal analysis of gene expression during aging in *Drosophila melanogaster* . Aging Cell 1:47–56. 12882353 10.1046/j.1474-9728.2002.00007.x

[evl389-bib-0074] Stearns, S. C. , M. Ackermann , M. Doebeli , and M. Kaiser . 2000 Experimental evolution of aging, growth, and reproduction in fruitflies. Proc. Natl. Acad. Sci. USA 97:3309–3313. 10716732 10.1073/pnas.060289597 PMC16235

[evl389-bib-0075] Stern, D. L. , and V. Orgogozo . 2008 The loci of evolution: how PredicDataset is genetic evolution? Evolution 62:2155–2177. 18616572 10.1111/j.1558-5646.2008.00450.x PMC2613234

[evl389-bib-0076] Suh, Y. , G. Atzmon , M. O. Cho , D. Hwang , B. Liu , D. J. Leahy et al. 2008 Functionally significant insulin‐like growth factor I receptor mutations in centenarians. Proc. Natl. Acad. Sci. USA 105:3438–3442. 18316725 10.1073/pnas.0705467105 PMC2265137

[evl389-bib-0077] Tatar, M. , A. Bartke , and A. Antebi . 2003 The endocrine regulation of aging by insulin‐like signals. Science 299:1346–1351. 12610294 10.1126/science.1081447

[evl389-bib-0078] Tricoire, H. , V. Battisti , S. Trannoy , C. Lasbleiz , A. M. Pret , and V. Monnier . 2009 The steroid hormone receptor EcR finely modulates *Drosophila* lifespan during adulthood in a sex‐specific manner. Mech. Age. Dev. 130:547–552. 10.1016/j.mad.2009.05.004 19486910

[evl389-bib-0079] Troemel, E. R. , S. W. Chu , V. Reinke , S. S. Lee , F. M. Ausubel , and D. H. Kim . 2006 p38 MAPK regulates expression of immune response genes and contributes to longevity in *C. elegans* . PLoS Genet. 2:e183. 17096597 10.1371/journal.pgen.0020183 PMC1635533

[evl389-bib-0080] Valanne, S. , J. H. Wang , and M. Rämet . 2011 The *Drosophila* Toll Signaling Pathway. J. Immunol. 186:649–656. 21209287 10.4049/jimmunol.1002302

[evl389-bib-0081] Willcox, B. J. , T. A. Donlon , Q. He , R. Chen , J. S. Grove , K. Yano et al. 2008 FOXO3A genotype is strongly associated with human longevity. Proc. Natl. Acad. Sci. USA 105:13987–13992. 18765803 10.1073/pnas.0801030105 PMC2544566

[evl389-bib-0082] Yunger, E. , M. Safra , M. Levi‐Ferber , A. Haviv‐Chesner , and S. Henis‐Korenblit . 2017 Innate immunity mediated longevity and longevity induced by germ cell removal converge on the C‐type lectin domain protein IRG‐7. PLoS Genet. 13:e1006577. 28196094 10.1371/journal.pgen.1006577 PMC5308781

[evl389-bib-0083] Zerofsky, M. , E. Harel , N. Silverman , and M. Tatar . 2005 Aging of the innate immune response in *Drosophila melanogaster* . Aging Cell 4:103–108. 15771614 10.1111/j.1474-9728.2005.00147.x

[evl389-bib-0084] Zhu, F. , H. Ding , and B. Zhu . 2013 Transcriptional profiling of *Drosophila* S2 cells in early response to *Drosophila* C virus. Virol. J. 10:210. 23803447 10.1186/1743-422X-10-210 PMC3704779

[evl389-bib-0085] Zwaan, B. J. 1999 The evolutionary genetics of ageing and longevity. Heredity 82:589–597. 10383679 10.1046/j.1365-2540.1999.00544.x

[evl389-bib-0086] Zwaan, B. , R. Bijlsma , and R. F. Hoekstra . 1995 Direct selection on life span in *Drosophila melanogaster* . Evolution 49:649–659. 28565142 10.1111/j.1558-5646.1995.tb02301.x

