# Supplementary material for: Evolution of longevity improves immunity in Drosophila
Source: Evol Lett. 2018 Nov 12;2(6):567–79. doi: 10.1002/evl3.89 (PMC6292704; doi:10.1002/evl3.89)
Supplement: Supplementary file 1 — Supplementary methods (pdf). Description of all methods, including details of selection and control lines, next‐generation sequencing, bioinformatic and statistical analyses, gene expression analyses, immunity assays, transgenic RNAi and lifespan assays. [file EVL3-2-567-s001.doc]

*Supplementary Methods*

**LONGEVITY SELECTION AND CONTROL LINES**

For our experiments we used lines from a long-term selection experiment for postponed reproduction (and thus indirectly for increased lifespan) in *Drosophila melanogaster*, first published in 1984 (Luckinbill et al. 1984); these selection lines are still being maintained under selection by one of us (R.A.). Details of this selection experiment and its maintenance are described in Luckinbill et al. (1984), Luckinbill and Clare (1985), Arking (1987), Arking et al. (2000a, b) and Buck and Arking (2001). In brief, an outbred base population was founded in 1979 from ~50 flies caught in a peach orchard in Michigan; the base population was then expanded, split into replicate lines, and selection was initiated in 1981. In each generation, selected L (long-lived') lines were propagated from eggs laid by relatively 'old' females (initially 22-day old females; after generation 25 from the 20-30% longest surviving females), whereas control R ('random bred') lines were propagated in a non-selective fashion by breeding flies at an adult age between 4 and 30 days, chosen in each generation by a random number generator (Arking 1987; Buck and Arking 2001). For our experiments we used 4 long-lived selection lines (La, Lb, 2La, 2Lb) and 2 unselected control lines (Ra, Rb). We performed genomic analysis of the selection lines after ≥144 generations of selection; at this point, control lines had been propagated for ≥ 293 generations. Prior to sequencing we reconfirmed the previously observed longevity phenotype of the selection lines: selected flies lived substantially longer than control flies (median female lifespan ≈ 62.5 days; percent lifespan extension relative to controls ≈ 38.8%; median male lifespan ≈ 72 days; percent lifespan extension relative to controls ≈ 50%; data not shown). Long-lived females were also less fecund early in life than control females (data not shown), indicating the existence of a trade-off between early fecundity and longevity, as previously reported (Luckinbill et al. 1984). Experimental assays were carried out after ≥162 generations of selection (control lines: ≥ 322 generations) (see below). Prior to assays, lines were kept as large populations (>2000 adults) in a two-week culture regime under relaxed selection. Flies were maintained and assays performed at 25°C, on a 12:12h light:dark cycle, and using a cornmeal-yeast-sucrose-agar diet (per 1 liter of food: 7 g agar, 50 g sucrose, 50 g cornmeal, 50 g yeast, 6 ml propionic acid, 10 ml of a 20% nipagin stock solution).

**NEXT-GENERATION POOL-SEQUENCING AND DATA PROCESSING**

To analyze the genomes of the selection and control lines we used Pool-Seq (Schlötterer et al. 2014). For each replicate line, we extracted DNA from pools of 100 females using the Qiagen DNeasy Blood and Tissue Kit and subsequent fragmentation with a Covaris S2 ultra sonicator (Covaris). Sequencing was performed by the Beijing Genomics Institute (BGI, Shenzhen, China). Whole-genome DNA libraries were prepared with the Paired-End DNA Sample Preparation Kit (Illumina); each sample was sequenced on two lanes on an Illumina HiSeq2000, generating 101 bp long paired-end reads. After trimming and filtering raw reads (minimum length: 50 bp, minimum average base quality: 18) with *PoPoolation* (Kofler et al. 2011a), we mapped sequences to the *D. melanogaster* genome v.5.4 and to *Wolbachia* (v.AE017196.1) with *bwa* (v.0.5.8c; Li and Durbin 2009) and removed duplicates with *Picard* (v.1.65; http://broadinstitute.github.io/picard/). We discarded all reads with a mapping quality lower than 20 and masked repeats and insertion/deletions (indels) with *RepeatMasker* (http://repeatmasker.org) and *PoPoolation2* (Kofler et al. 2011b), respectively. After file processing, the average genomic coverage was ~120-260x. Sites with a coverage lower than 15x and higher than 2% of maximum coverage were excluded for analysis. We restricted our analysis to the normally recombining regions of the genome: *X*: 1,036,552-20,902,578; *2L*: 844,225-19,946,732; *2R*: 6,063,980-20,322,335; *3L*: 447,386-18,392,988; *3R*: 7,940,899-27,237,549 (as defined in reference Kolaczkowski et al. 2011). All selection and control lines were infected with *Wolbachia*, and there were no consistently differentiated *Wolbachia* loci between selection and control lines (results not shown); it thus unlikely that our results are confounded by systematic variation due to differential *Wolbachia* infection among lines / the selection regimes.

**DEFINITION OF CANDIDATE SNPs AND GENES**

To define candidate SNPs that are likely shaped by selection we applied a highly stringent *F*_ST_ outlier approach (Akey 2009; Lewontin and Krakauer 1973) to all 1,307,590 polymorphic sites, using *PoPoolation2* (Kofler et al. 2011b). Alleles occurring less than 12 times across all six lines (average minimum count of 2 per line) were excluded. SNPs were deemed to represent candidates if their frequencies were (i) strongly and consistently differentiated between the selection and control regimes, with an *F*_ST_ > 0.9 in all 8 possible pairwise comparisons of the 2 control lines with the 4 selection lines (Fig. 1a) and (ii) significantly different between regimes in a Fisher's exact test (at α= 0.001) with Bonferroni correction (p < α' = 0.001 / 1,307,590 = 7.65 x 10^-10^). This yielded 8205 candidate SNPs in 868 genes. In addition to defining candidates, we estimated the strength of differentiation among selection versus control lines relative to differentiation within selection or control lines by calculating a ‘selection signal-to-noise’ ratio for each polymorphic SNP, with the ratio ranging from 1 to -1 (see Fig. 1b):


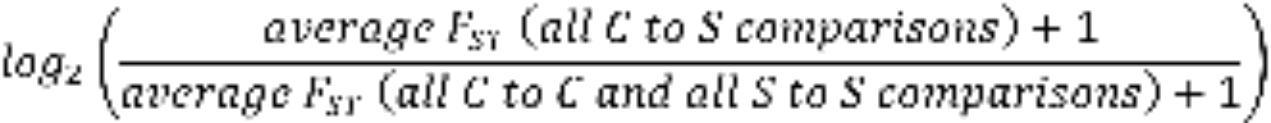


where C refers to control lines and S to selection lines. For maximal differentiation among selection versus control lines at *F*_ST_ = 1, i.e. with no differentiation within selection or control lines, this ratio becomes log_2_ (2/1) = 1. For minimal differentiation among selection versus control lines, but maximal differentiation among lines within regimes, the ratio gives log_2_ (1/2) = -1. When the amount of differentiation is the same among regimes as within regimes then the ratio is log_2_ (1/1) = 0. To confirm that our final list of candidates is not the result of chance (e.g., due to genetic drift or bottlenecks) we applied the above candidate criteria to all possible combinations of 8 pairwise comparisons. With our design there are 15 possible pairwise comparisons between distinct lines (8 control-selection, 6 selection-selection, and 1 control-control comparisons). Thus, in total there exist 6435 (
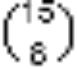
) possible combinations of 8 pairwise comparisons, but only one set of 8 pairwise control-selection comparisons is biologically informative in terms of inferring selection, i.e. consistent differentiation between the control versus selection lines. No combination of 8 pairwise comparisons yielded as many candidate SNPs as the ‘true’ set of 8 control-selection comparisons, with a probability that the ‘true’ number of candidate SNPs is due to chance of p ≈ 1.6 x 10^-4^ (see Fig. 1d).

**DOWNSTREAM ANALYSES OF CANDIDATES**

We performed a number of downstream analyses on our candidate set of SNPs. First, to identify differences in the proportion of genomic features of candidates versus whole-genome background, we obtained gene names and annotations for the candidates from FlyBase r.5.40 with *SnpEff* (v.2.0.3; Cingolani et al. 2012). To account for potential regulatory variants up- or downstream of the gene body we considered all sequence variants within 1 kb distance of the 5’ and 3’UTR as being part of a given candidate gene; variants outside of these borders were considered to be intergenic. The analyzed ‘candidate gene’ sequences thus include both synonymous and non-synonymous SNPs as well as exons and introns. Second, to examine the functions of the candidate genes and test for gene set enrichment we used gene ontology (GO) analysis in *Gowinda* (Kofler and Schlötterer 2012), accounting for gene length bias. Genes were mapped to GO terms with *GoMiner* (Zeeberg et al. 2003), using the reference genome v.5.40 for annotations. We considered the ontologies ‘Biological Function’, ‘Molecular Function’ and ‘Cellular Component’. Finally, to investigate ‘gene reuse’, i.e. the extent to which genetic loci underlying the evolution of *Drosophila* lifespan might be shared (‘public’) or lineage/population-specific (‘private’) (DeVeale et al. 2004; Partridge and Gems 2002), we compared our list of candidate genes to those from two 'Evolve and Resequence' (E&R) studies (Carnes et al. 2015; Remolina et al. 2012) similar to ours (Luckinbill et al. 1984). To do so, we first created uniform gene names using the Flybase Upload/Convert IDs tool (v.FB2014_03; http://flybase.org/convert/id) and then calculated all possible overlaps between the three gene lists using the R package *SuperExactTest* (Wang et al. 2015), assuming a shared background of 12,007 genes in the normally recombining genome (Kolaczkowski et al. 2011, also cf. Table S5).

**ANALYSIS OF GENE EXPRESSION**

As a first functional test of the observed genetic differentiation in immunity genes between selection and control lines, we measured the expression of three antimicrobial peptides (AMPs), *Drosomycin* (*Drs*), *Attacin A* (*AttA*) and *Diptericin* (*Dpt*). We assayed expression of young (5-6-day-old) and aged (25-26-day-old) female flies, either without pricking (baseline expression), upon aseptic pricking with a sterile needle (mock infection control) or upon prick infection with *Erwinia carotovora carotovora 15* (*Ecc 15*). While we did not identify these AMPs as longevity candidates, they serve as robust readouts of Toll and Imd activity (De Gregorio et al. 2002; Lemaitre et al. 1997; Neyen et al. 2014). *Drs* and *AttA* are regulated by both Imd and Toll signaling, and *Dpt* is predominantly regulated by the Imd pathway (De Gregorio et al. 2002). Infections with *Ecc15* cause the induction of all three AMPs mentioned above (Basset et al. 2000; De Gregorio et al. 2002; Lemaitre et al. 1997). We assayed expression levels 4-6 hours after treatment (uninfected = no treatment; pricked = mock control; infected). For each line, age and treatment (uninfected; pricked; infected), we performed qRT-PCR analysis on three biological replicates, each with three technical replicates. For each biological replicate, we homogenized five female flies with a pestle rotor. RNA was extracted with the MagMAX Total RNA Isolation Kit (Ambion) on a MagMAX Express Magnetic Particle Processor (Applied Biosystems), followed by cDNA synthesis with the GoScript Reverse Transcription System (Promega). qRT-PCR was performed with the SYBR Green-based GoTaq qPCR Mix (Promega) on a QuantStudio 6 Flex Real-Time PCR System (Applied Biosystems). Efficiency-corrected Ct values of AMP genes were normalized to the geometric mean expression of two reference genes *Rp49* (*RpL32*) and *Gapdh2*. We used the following forward (F) and reverse (R) primer pairs:

*AttA* F: 5’-CCCGGAGTGAAGGATG-3’;

*AttA* R: 5’-GTTGCTGTGCGTCAAG-3’;

*Dpt* F: 5’-GCTGCGCAATCGCTTCTACT-3’;

*Dpt* R: 5’- TGGTGGAGTGGGCTTCATG-3’;

*Drs* F: 5’-CGTGAGAACCTTTTCCAATATGAT-3’;

*Drs* R: 5’-TCCCAGGACCACCAGCAT-3’;

*Rp49* F: 5’-AATGATGTGCGAGTGCCGAG-3’;

*Rp49* R: 5’-CAATGGTGCTGCTATCCCAATC-3’

*Gapdh2* F: 5’-GCGGTAGAATGGGGTGAGAC-3’;

*Gapdh2* R: 5’-TGAAGAGCGAAAACAGTAGC-3’.

Expression data (i.e., log_10_ (relative baseline expression levels of uninfected flies) or expression ratios (infected/mock)) were analyzed with ANOVA (expression = Regime + Age + Age x Regime) in JMP v.10.0 (SAS Institute Inc.). For baseline expression levels, we also estimated the random effect of Line(Regime) with REML. This effect was included to account for variation among lines within a regime; since this effect is not of primary interest we do not report variance component estimates. In contrast, for expression ratios, Line(Regime) was the lowest level of replication and could not be estimated. Sample sizes and details of statistical tests are given in Table S5.

**INFECTION ASSAYS**

To examine whether selected and control lines differ in their realized immune response, we performed infection assays, following standard procedures (Neyen et al. 2014). We measured adult female survival upon infection with four different pathogens at two different ages (young females: 1-4-days-old; aged females: 22-25-days-old), except for infections with *Drosophila* C virus (DCV) where only young flies were used. Infections with the entomopathogenic fungus *Beauveria bassiana* were performed by shaking and rolling CO_2_ -anaesthetized flies in a dish containing sporulating fungi until all flies were covered in spores. For systemic infections with bacteria, single colonies of *Enterococcus faecalis* (gram-positive) and *Erwinia carotovora carotovora* (*Ecc15*, gram-negative) were cultured in Luria Broth (LB) medium for ~24 hours under constant shaking at 37°C and 29°C, respectively. Flies were infected under CO_2_ anesthesia by pricking the thorax with a fine needle (tip diameter 0.15 mm, Fine Science Tools) dipped into bacterial solution (OD_600_ = 8 for *E. faecalis*; OD_600_ = 200 for *Ecc15).* Infections with DCV (courtesy of Luis Teixeira, IGC, Oeiras, Portugal) were performed by pricking 1-4-days-old flies with a needle dipped into viral suspension (2x10^7^ particles / ml = TCID_50_). For all infection assays, flies were transferred to fresh vials every second day and survival was recorded daily. Survival assays were terminated 25 days post-infection, except for the DCV assay which was terminated after 13 days; for statistical analysis, survival data were right-censored from day 7 onwards (and from day 14 for the assay with *B. bassiana*). Mortality data were analyzed using Cox (proportional hazards) regression with *χ*^2^ tests in JMP v.10.0 (SAS Institute Inc.). For details of sample sizes and statistical tests see Table S5.

**BACTERIAL CLEARANCE ASSAY**

To test whether the selection and control lines differ in their clearance ability, we estimated the proportion of flies that successfully managed to completely clear an infection. We infected mated females at two ages (young: 5-6-days old; aged: 23-25-days old) with a bacterial solution containing Rifampicin-resistant *Ecc15* (OD_600_=200). Over a period of 6 days post-infection, we harvested 10 live flies per line per day and homogenized each fly in liquid LB medium. 3 µl of each homogenate and of three dilutions per homogenate (1:10, 1:100, 1:1000) were spotted in duplicate on LB plates containing 100 µg/ml Rifampicin (Sigma Aldrich) to selectively amplify the Rifampicin-resistant *Ecc15* used for infection. After 18 hours of incubation at room temperature, we counted the number of spots without any bacterial growth and determined the proportion of flies that had completely cleared the infection. Data were analyzed with a binomial GLM (model: proportion full clearance = Regime + Age + Regime x Age) in JMP v.10.0 (SAS Institute Inc.). Line(Regime) was the lowest level of replication in this analysis and could thus not be estimated. For sample sizes and statistical tests see Table S5.

**RNAI AND LIFESPAN ASSAYS**

To test whether candidate genes in the Toll pathway affect lifespan, we employed transgenic RNAi in adult flies. We used a ubiquitously expressing, mifepristone (RU486)-inducible *daughterless(da)*-GeneSwitch(GS)-GAL4 (Tricoire et al. 2009) (courtesy of Véronique Monnier, Paris) to drive expression of the following UAS-RNAi constructs obtained from the Vienna *Drosophila* RNAi Center (VDRC) or the Bloomington *Drosophila* Stock Center (BDSC): UAS-*Spätzle (spz*)-RNAi (VDRC #105017); UAS-*Toll*(*Tl*)-RNAi (VDRC #100078); UAS-*cactus*(*cact*)-RNAi (BDSC #34775); and UAS-*Dorsal-related immunity factor* (*dif*)-RNAi (BDSC #30513). Cohorts of F1 offspring between crosses of *da-*GS-GAL4 virgin females and males carrying UAS-RNAi constructs were collected within a 24-hour window; 24 hours after eclosion, flies were sexed, genotyped under mild CO_2_ exposure and transferred to 1-liter demography cages. 1-2 day old experimental flies were either fed medium containing 200 µg/ml (466 µM) mifepristone dissolved in ethanol (see Scialo et al. 2016) to induce *da-*GS-GAL4>UAS-RNAi or control medium (uninduced, ethanol without mifepristone), using food vials attached to the cages. For each genotype and diet, we set up three replicate cages, each containing on average ~ 72 flies per sex. In addition, we included three replicate cages with F1 flies from a cross between *da*-GS-GAL4 and *w^1118^* (BDSC #3605) to control for the potential effects of mifepristone treatment upon lifespan; we did not find any confounding effects of mifepristone on lifespan (see Table S5). Dead flies were scored and fresh food was provided every two days. Differences in lifespan between mifepristone-induced RNAi and uninduced controls were analyzed using mixed-effects Cox (proportional hazards) regression with 'Treatment' (mifepristone vs. ethanol), 'Sex' and the 'Treatment x Sex' interaction as fixed effects and with 'Cage' as a random effect using the R package coxme. Escaped flies were censored. See Table S5 for details of sample sizes and statistical tests.

**DATA AVAILABILITY**

Sequencing data used for analyses are available from the European Nucleotide Archive (ENA) under accession PRJEB28048 / ERP110212; raw data for experimental assays are available from Dryad under accession *TO BE ADDED UPON ACCEPTANCE*.

**REFERENCES**

Arking, R. (1987). Successful selection for increased longevity in *Drosophila*: Analysis of the survival data and presentation of a hypothesis on the genetic regulation of longevity. *Exp. Gerontol.* 22:199–220.

Arking, R., Burde, V., Graves, K., Hari, R., Feldman, E., Zvei, A. et al. (2000a) Forward and reverse selection for longevity in *Drosophila* is characterized by alteration of antioxidant gene expression and oxidative damage patterns. *Exp. Gerontol*. 35:167-185.

Arking, R., Burde, V., Graves, K., Hari, R., Feldman, E., Zvei, A. et al. (2000b). Identical longevity phenotypes are characterized by different patterns of gene expression and oxidative damage. *Exp. Gerontol*. 35:353-373.

Basset, A., Khush, R.S., Braun, A., Gardan, L., Boccard, F., Hoffmann, J.A. et al. (2000). The phytopathogenic bacteria *Erwinia carotovora* infects *Drosophila* and activates an immune response. *Proc. Natl. Acad. Sci.* *USA* 97:3376-3381.

Buck, S.A., and Arking, R. (2001). Metabolic alterations in genetically selected *Drosophila* strains with different longevities. *J. Am. Aging Assoc*. 24:151-161.

Carnes, M.U., Campbell, T., Huang, W., Butler, D.G., Carbone, M.A., Duncan, L.H. et al. (2015). The Genomic Basis of Postponed Senescence in *Drosophila melanogaster*. *PLoS One* 10:e0138569.

Cingolani, P., Platts, A., Wang Ie, L., Coon, M., Nguyen, T., Wang, L. et al. (2012). A program for annotating and predicting the effects of single nucleotide polymorphisms, SnpEff. *Fly* 6:80-92.

De Gregorio, E., Spellman, P.T., Tzou, P., Rubin, G.M., and Lemaitre, B. (2002). The Toll and Imd pathways are the major regulators of the immune response in *Drosophila*. *EMBO J.* 21:2568-2579.

Kofler, R., Orozco-terWengel, P., De Maio, N., Pandey, R.V., Nolte, V., Futschik, A. et al. (2011a). PoPoolation: A Toolbox for Population Genetic Analysis of Next Generation Sequencing Data from Pooled Individuals. *PLoS ONE* 6:e15925.

Kofler, R., Pandey, R.V., and Schlötterer, C. (2011b). PoPoolation2: Identifying differentiation between populations using sequencing of pooled DNA samples (Pool-Seq). *Bioinformatics* 27:3435–3436.

Kofler, R., and Schlötterer, C. (2012). Gowinda: Unbiased analysis of gene set enrichment for genome-wide association studies. *Bioinformatics* 28:2084-2085.

Kolaczkowski, B., Kern, A.D., Holloway, A.K., and Begun, D.J. (2011). Genomic differentiation between temperate and tropical Australian populations *of Drosophila melanogaster*. *Genetics* 187:245-260.

Lemaitre, B., Reichhart, J-M., and Hoffmann, J.A. (1997). *Drosophila* host defense: Differential induction of antimicrobial peptide genes after infection by various classes of microorganisms. *Proc. Natl. Acad. Sci. USA* 94:14614-14619.

Li, H., and Durbin, R. (2009). Fast and accurate short read alignment with Burrows-Wheeler transform. *Bioinformatics* 25:1754-60.

Luckinbill, L., and Clare, M. (1985). Selection for life span in *Drosophila melanogaster*. *Heredity* 55:9-18.

Luckinbill, L.S., Arking, R., Clare, M.J., Cirocco, W.C., and Buck, S.A. (1984). Selection for Delayed Senescence in *Drosophila melanogaster*. *Evolution* 38:996-1003.

Neyen, C., Bretscher, A.J., Binggeli, O., and Lemaitre, B. (2014). Methods to study *Drosophila* immunity. *Methods* 68:116-128.

Remolina, S.C., Chang, P.L., Leips, J., Nuzhdin, S.V., and Hughes, K.A. (2012). Genomic Basis of Aging and Life History Evolution in *Drosophila melanogaster*. *Evolution* 66:3390-3403.

Scialo, F., Sriram, A., Stefanatos, R., & Sanz, A. (2016). Practical Recommendations for the Use of the GeneSwitch Gal4 System to Knock-Down Genes in *Drosophila melanogaster*. *PLoS One* 11(8):e0161817.

Tricoire, H., Battisti, V., Trannoy, S., Lasbleiz, C., Pret, A.M., and Monnier, V. (2009). The steroid hormone receptor EcR finely modulates *Drosophila* lifespan during adulthood in a sex-specific manner. *Mech. Age. Dev.* 130:547-552.

Wang, M., Zhao, Y., and Zhang, B. (2015). Efficient Test and Visualization of Multi-Set Intersections. *Sci. Rep.* 5:16923.

Zeeberg, B.R., Feng, W., Wang, G., Wang, M.D., Fojo, A.T., Sunshine, M., et al. (2003). GoMiner: a resource for biological interpretation of genomic and proteomic data. *Genome Biol.* 4:R28.
